# LMP-420, a small-molecule inhibitor of TNF-alpha, reduces replication of HIV-1 and *Mycobacterium tuberculosis *in human cells

**DOI:** 10.1186/1742-6405-3-8

**Published:** 2006-03-31

**Authors:** Soichi Haraguchi, Noorbibi K Day, Wasu Kamchaisatian, Macarena Beigier-Pompadre, Steffen Stenger, Nutthapong Tangsinmankong, John W Sleasman, Salvatore V Pizzo, George J Cianciolo

**Affiliations:** 1 Department of Pediatrics, University of South Florida, 801 Sixth Street South, St. Petersburg, FL 33701, USA; 2 Institut für Klinische Mikrobiologie, Immunologie und Hygiene der Friedrich-Alexander Universität Erlangen-Nürnberg, Erlangen, Germany; 3 Department of Pathology, Duke University Medical Center, Durham, NC 27710, USA

## Abstract

**Background:**

Co-infections of human immunodeficiency virus (HIV) and *Mycobacterium tuberculosis *(*M. Tb*) are steadily increasing and represent a major health crisis in many developing countries. Both pathogens individually stimulate tumor necrosis factor-alpha (TNF) release from infected cells and TNF, in turn, enhances the replication of each. A recent report on a Phase I clinical trial suggested that etanercept (soluble TNF receptor) might be beneficial in treating HIV/*M. Tb *co-infected patients. We sought to determine if a small molecule inhibitor of TNF synthesis and activity could block replication of either organism and thus be a potential adjunct to existing drugs targeting these agents.

**Results:**

LMP-420, a novel anti-inflammatory agent that inhibits TNF, was tested for HIV-1 inhibition both alone and in combination with AZT (3' -azido-3-deoxythymidine). LMP-420 alone was tested against *M. Tb*. HIV-1 infected human peripheral blood mononuclear cells (PBMC) or *M. Tb*-infected human alveolar macrophages (AM) were treated with a single dose of LMP-420 and viral or bacterial replication determined after 7 or 5 days respectively. Viral replication was determined from supernatant p24 levels measured by ELISA. *M. Tb *replication was determined by bacterial culture of macrophage lysates. LMP-420 alone inhibited HIV replication over 7 days with an IC_50 _of ~300 nM. Combination of LMP-420 with AZT doubled the level of HIV inhibition observed with AZT alone. LMP-420 alone inhibited the replication of virulent *M. Tb *by >80%, more than that observed with anti-TNF antibody alone.

**Conclusion:**

Inhibition of TNF with inexpensive, small-molecule, orally-active drugs may represent a useful strategy for enhancing the activity of currently-available antiviral and anti-*M. Tb *agents, particularly in those areas where co-infections with these pathogens act to synergistically enhance each other.

## Background

AIDS and tuberculosis annually kill more than three million people worldwide and the numbers are growing. Of the >40 million adults and 5 million children infected with HIV, 95 percent live in developing countries and about one-third are co-infected with *M. Tb*. As many as half of HIV-infected patients in Africa have *M. Tb *and up to 80 percent of *M. Tb*-infected patients are infected with HIV. People co-infected with both HIV and *M. Tb *have a 100-fold greater risk of developing active *M. Tb *disease and becoming infectious, increasing the spread of disease even further and faster. If active *M. Tb *goes untreated in HIV+ patients, most will die within one year.

*M. Tb *is the most common opportunistic infection occurring in HIV-infected individuals in resource poor countries and it accelerates HIV-associated morbidity and mortality as well as viral replication [1]. Studies have shown increased transcription of the HIV long terminal repeat (LTR) in cultured monocytic cells exposed to either live *M. Tb *or cell wall components [2]. In these same studies anti-TNF antibodies reduced the increased transcription of the HIV LTR by >50% [2]. Kitaura *et al*. [3] demonstrated that incubation of U1, a chronically HIV-infected human promonocytic cell line, with various strains of mycobacteria resulted in enhanced p24 antigen release into the supernatant. The amount of TNF produced by U1 cells correlated with p24 antigen release and antibody to TNF inhibited p24 release induced by mycobacteria. In a recent review Collins *et al*. [4] postulate that higher viral loads, increased HIV diversity, and changes in cytokine/chemokine levels in HIV-infected individuals with *M. Tb *appear to be related to a localized immune stimulation. They suggest that increased levels of TNF and MCP-1, induced by *M. Tb*, may activate HIV replication in lymphocytes, monocytes, and macrophages that are resident or have migrated to *M. Tb*-infected organs, such as the pleura or lung. In addition, studies from the same group demonstrated that the HIV present in blood, following a *M. Tb*-mediated burst in load and diversity, is phylogenetically related to HIV clones that have evolved independently in *M. Tb*-infected lung or pleural compartments [5]. The potential of MCP-1 (CCL2) to upregulate HIV replication was also confirmed by Fantuzzi *et al*. [6] who reported that infection of monocyte-derived macrophages with laboratory-adapted HIV or primary viral isolates in the continuous presence of anti-CCL2 antibody resulted in significantly lower p24 release compared to control cultures. Further, CCL2 neutralization resulted in the intracellular accumulation of p24 antigen and they suggested that CCL2 might represent an autocrine factor important for enhancing virion production, most likely by affecting the macrophage cytoskeleton.

Other organisms also enhance HIV replication through increased TNF production. Zhao *et al*. [7] reported that the protozoan parasite *Leishmania *enhances both HIV virus transcription and production in human tonsillar tissue infected *ex vivo*. Use of pentoxifylline and neutralizing anti-TNF or anti-IL-1-alpha antibodies showed that this *Leishmania*-mediated increase in HIV production was linked to increased production of TNF and IL-1-alpha.

As noted above, TNF-mediated enhancement also applies to the replication of *M. Tb*. Engele *et al*. [8] demonstrated that infection of human alveolar macrophage (AM) with virulent strains of mycobacteria induced the secretion of significantly higher levels of TNF than attenuated strains and that TNF levels correlated with the ability of the mycobacteria to multiply intracellularly. Treatment of infected macrophages with anti-TNF antibodies reduced, while exogenously-added TNF enhanced, the growth rate of intracellular bacteria.

Studies supporting the potential role of TNF in HIV replication and pathogenesis are those of De *et al*. [9], who used HIV-transgenic mice (tg26) which appear normal at birth but die within 3- 4 weeks. The skin of these transgenic mice shows diffuse scaling and expresses high levels of both HIV mRNA and gp120. Sera of Tg26 mice have a 50-fold increase in TNF levels compared to those of non-transgenic mice. Treatment with antibody to TNF reduced serum TNF levels by ~75%, prevented death, resulted in near normal growth, and produced a marked decrease in skin lesions and a profound reduction in the expression of HIV mRNA and gp120. Sha *et al*. [10] reported the results of a clinical study in which etanercept (Enbrel^®^; dimerized soluble TNF receptor) was used for as a single bolus to treat HIV-infected subjects who had already received 12 weeks of HAART (highly active antiretroviral therapy) followed by an additional 16 weeks of HAART with or without recombinant human interleukin-2 (rhIL-2). Plasma IL-6 and C-reactive protein levels increased after rhIL-2 treatment but etanercept pretreatment blunted these increases and appeared to be well tolerated. Recently, Wallis *et al*. [11] reported on a 28-day Phase I safety study of etanercept in 16 patients co-infected with HIV-1 and *M. Tb*. Etanercept (25 mg) was administered twice weekly, beginning on day 4 of the *M. Tb *therapy, for 4 weeks. Controls were 42 CD4-frequency-matched patients with sputum smear-positive *M. Tb *and CD4 cell counts >200 cells/μl. In etanercept-treated subjects trends toward superior responses to *M. Tb *treatment were evident in body mass, performance score, number of involved lung zones, cavitary closure, and time to sputum culture conversion. Etanercept treatment resulted in a 25% increase in CD4 cells by week 4 although none of these positive trends were statistically significant because of the low number of patients enrolled in this Phase I study.

Thus, several studies suggest that minimizing TNF levels in both HIV and *M. Tb*-infected patients might decrease both the replication of each pathogen as well as the pathogenesis associated with co-infection of these two agents. Furthermore, pilot clinical studies suggest that TNF levels can be safely lowered in conjunction with existing antiretroviral or anti-*M. Tb *therapies. Currently-marketed TNF antagonists are proteins requiring injection and with relatively long half-lives, making it more difficult to cease TNF antagonism once it has been initiated. In the present study we sought to determine whether LMP-420, an anti-inflammatory nucleoside analog that is a potent inhibitor of TNF and MCP-1 synthesis, would block the replication of either HIV or virulent *M. Tb *in human primary cell cultures and whether LMP-420 might synergize with AZT to inhibit HIV replication.

## Results

### Inhibition by LMP-420 of TNF secretion by LPS-stimulated PBMC

**Figure 1:**
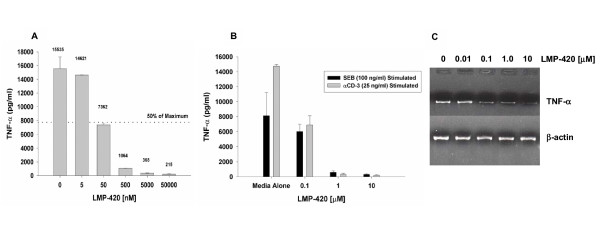
Inhibition of TNF by LMP-420. A) Dose response of LMP-420 on human PBMC stimulated with LPS. PBMC were resuspended to 3.75 × 10^6 ^total cells/ml in complete RPMI 1640 medium (containing 5% heat-inactivated human AB serum) and 0.4 ml of cell suspension put into each well of a 48-well tissue culture plate. To each well was added 0.1 ml of media or media containing LMP-420 (diluted from a stock solution of 338 mM in DMSO) to give the indicated final concentration. The cell cultures were incubated for 2 h at 37°C in humidified 5% CO_2 _and then 55 μl of media or LPS (*S. typhosa*, 1 μg/ml of media) was added to each well. The cultures were incubated 20 h at 37°C, the contents of each well removed to a 5-ml polypropylene centrifuge tube and centrifuged for 20 min at 400 g. The supernatants were removed to a fresh tube and frozen at -20°C until assayed by solid phase ELISA (R & D Systems). B) Dose response of LMP-420 on human PBMC stimulated with anti-CD3 or SEB. PBMC were resuspended to 3.75 × 10^6 ^total cells/ml in complete RPMI 1640 medium (containing 5% heat-inactivated human AB serum) and 0.4 ml of cell suspension put into each well of a 48-well tissue culture plate. To each well was added 0.1 ml of media or media containing LMP-420 (diluted from a stock solution of 338 mM in DMSO) to give the indicated final concentration. The cell cultures were incubated for 2 h at 37°C in humidified 5% CO_2 _and then 55 μl of media or anti-CD3 (25 ng/ml final concentration) or SEB (100 ng/ml final concentration) was added to each well. The cultures were incubated for 48 h at 37°C, the contents of each well removed to a 5-ml polypropylene centrifuge tube and centrifuged for 20 min at 400 g. The supernatants were removed to a fresh tube and frozen at -20°C until assayed by solid phase ELISA (R & D Systems). C) RT-PCR of samples prepared from LPS-stimulated human PBMC. PBMC (1 × 10^6^/well) were incubated for 2 h at 37°C with the indicated concentration of LMP-420 and then stimulated for 3 h at 37°C with LPS (*S. typhosa*; 1 μg/ml). Cells were harvested, total RNA extracted, cDNA prepared and RT-PCR performed for 30 cycles using primers for TNF-α and β-actin obtained from R & D Systems.

Figure 1A illustrates inhibition of TNF accumulation in supernatants of human PBMC cultured for 20 h with 1 μg/ml of LPS after a 2-h pretreatment with varying concentrations of LMP-420. LMP-420 inhibits LPS-stimulated TNF release in a dose-dependent manner with an IC_50 _of approximately 50 nM. At the highest concentration tested (50 μM), LMP-420 inhibited 98.6% of TNF release compared to a DMSO alone (0.5%) control. Since TNF released from LPS-stimulated PBMC is derived mostly from monocytes/macrophages, we wanted to determine if LMP-420 could also inhibit TNF release from stimulated lymphocytes. As shown in Figure 1B, LMP-420 also inhibited TNF release from PBMC stimulated with either of two different lymphocyte-stimulating reagents, monoclonal anti-CD3 antibody or SEB.

### LMP-420 inhibits TNF at the transcriptional level

Inhibition of TNF release can occur at a number of different points in stimulated cells. These might include initial binding of the stimulating ligand, transcription of TNF mRNA, translation of mRNA to protein, expression of TNF on cell membranes, or the cleavage of TNF from the cell membrane. Since LMP-420 inhibits TNF release from PBMC stimulated with a variety of ligands other than LPS (including IL-2, zymosan, Pansorbin; data not shown) we assumed that inhibition was occurring at a post-stimulus step. PBMC were treated for 2 h with different concentrations of LMP-420, the cells stimulated an additional 3 h with LPS, total mRNA isolated, cDNA prepared, and RT-PCR performed using specific primers for human TNF. As shown in Figure 1C, LMP-420 inhibits transcription of mRNA for TNF in a dose-dependent manner, suggesting that this is the mechanism by which it effects its inhibition of TNF protein release.

### LMP-420 is highly selective for TNF

In order to determine whether LMP-420 was for selective for inhibition of TNF, PBMC were incubated with either media or a single concentration of LMP-420 (1 μM; 20 × IC_50 _for inhibition of TNF) for 2 h and then incubated overnight with LPS (1 μg/ml). Supernatants were assayed for released cytokines/chemokines using a Bio-Rad multiplex assay kit which measures 17 cytokines/chemokines simultaneously. As shown in Table 1, LMP-420 potently inhibits the release of TNF and MCP-1 (91 and 95% respectively) with a lesser effect on the release of IL-1 and IFN-γ (33 and 38% respectively). In contrast, there was no significant effect on the levels of IL-6, IL-8, IL-10, G-CSF, or MIP-1β. Although there were slight (~30% or less) decreases in the levels of several other cytokines/chemokines, the levels present were too low to ascribe significance to these effects. Since these culture supernatants were from overnight cultures we cannot at this time rule out the possibility that the inhibition of MCP-1 is secondary to the inhibition of TNF by LMP-420, since TNF can itself stimulate release of MCP-1. This pattern of cytokine/chemokine inhibition is, of course, only representative of PBMC stimulated with a TLR4 ligand (LPS) which preferentially stimulates monocytes/macrophages and a similar pattern of cytokine/chemokine inhibition may not necessarily be observed with other stimuli.

**Table 1:** Selective inhibition of TNF-alpha by LMP-420

CYTOKINE	LPS-Stimulated Release (pg/ml)		Percent Inhibition
	
	Media Alone	+ LMP-420 (1 μM)	
IL-1β	15, 829 ± 75	10,670 ± 271	33
IL-2	24 ± 0	18 ± 1	25
IL-4	582 ± 54	419 ± 13	28
IL-5	7 ± 3	3 ± 1	57
IL-6	16,512 ± 190	17,249 ± 103	0
IL-7	26 ± 1	24 ± 1	8
IL-8	17,160 ± 221	17,575 ± 262	0
IL-10	1,963 ± 17	1,766 ± 62	10
IL-12	51 ± 3	76 ± 4	0
IL-13	44 ± 2	33 ± 0	25
IL-17	80 ± 1	56 ± 1	30
G-CSF	2,861 ± 98	4,794 ± 803	0
GM-CSF	93 ± 6	63 ± 4	32
MCP-1	2,714 ± 132	143 ± 0	95
MIP-1β	19,554 ± 63	19,127 ± 520	2
IFN-γ	424 ± 4	262 ± 1	38
TNF-α	7,628 ± 112	654 ± 7	91

### Inhibition of HIV-1 replication in human PBMC using LMP-420

**Figure 2:**
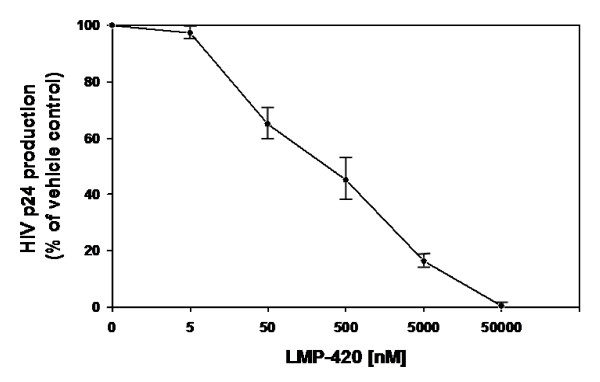
HIV-1_Ba-L _replication in PHA-stimulated human PBMC treated with indicated concentration of LMP-420. PBMC (10^6^/ml) were infected with HIV-1_Ba-L _for 2 h, treated with LMP-420 at the concentration indicated and incubated under the conditions described in *Methods*. The supernatants were harvested and stored at -80°C for HIV-1 p24 determinations as described in *Methods*. Data represent the average ± SEM of thee experiments using 3 different donors.

Because of LMP-420's ability to inhibit TNF release, both from monocytes/macrophages and T lymphocytes, we hypothesized that LMP-420 should inhibit the replication of HIV-1 since HIV-1 can induce TNF and TNF can activate HIV-1 viral LTR. Activated PBMC were incubated with varying concentrations of LMP-420, ranging from 0- 50000 nM, at the same time that HIV was added and then incubated for 7 days without further addition of LMP-420. As shown in Figure 2, LMP-420 inhibits in a dose-dependent manner the replication, as determined by HIV p24 antigen release, of HIV in PBMC. The percent of p24 release, compared to media controls, is 97%, 65%, 45%, 16% and 0.2% at LMP-420 concentrations of 5, 50, 500, 5000, and 50000 nM, respectively. Thus, at an LMP-420 concentration of 500 nM, HIV replication is inhibited by 55%. This same concentration of LMP-420 would inhibit ~90% or more of 24 h TNF release from LPS-stimulated PBMC (Figure 1A) and perhaps slightly less in cultures stimulated with lymphocyte-specific stimuli (Figure 1B).

### LMP-420 enhances the inhibitory effect of AZT on viral replication

**Figure 3:**
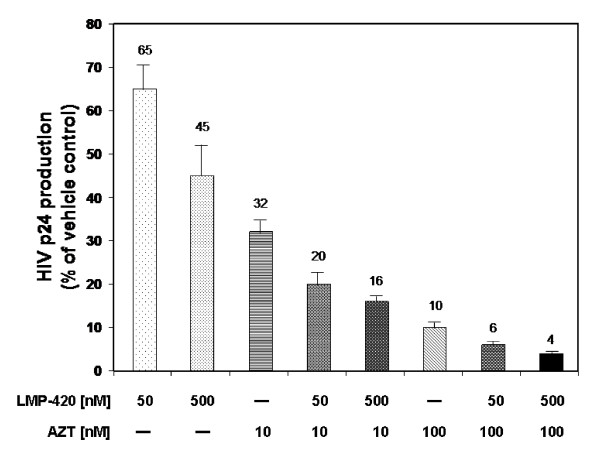
Effects of LMP-420 and/or zidovudine (AZT) on HIV-1 replication. PBMC (10^6^/ml) were infected with HIV-1_Ba-L _for 2 h, treated with LMP-420 and/or AZT at concentration indicated, and incubated under condition described in *Methods*. The supernatants were harvested and stored at -80°C for HIV-1 p24 determinations as described in *Methods*. Data represent the average ± SEM of thee experiments using 3 different donors.

Our hypothesis was that the LMP-420 inhibition of HIV replication shown in Figure 2 was due to the host cells' inability to support that replication through TNF release and subsequent activation of viral LTR in an autocrine or paracrine fashion. Based on this postulated mechanism, LMP-420 should synergize with antiviral agents, such as AZT, which target the virus directly. As shown in Figure 3, addition of suboptimal doses of 50 or 500 nM LMP-420 to a suboptimal dose of 10 nM AZT results in a further decrease of HIV-1 p24 antigen release from 32% (AZT alone) to 20% (AZT + 50 nM LMP-420) and 16% (AZT + 500 nM LMP-420) of controls. Addition of 50 or 500 nM LMP-420 to 100 nM AZT resulted in a decrease of HIV-1 p24 production from 10% (AZT alone) to 6% (AZT + 50 nM LMP-420) and 4% (AZT + 500 nM LMP-420) of vehicle control. Thus 500 nM LMP-420, a dose which by itself results in a 55% inhibition of viral replication, used in combination with AZT (10 nM or 100 nM) increases the inhibition of viral replication by 50% and 60% respectively when compared with AZT alone.

### LMP-420 inhibits the replication of virulent M. Tb in human AM

**Figure 4:**
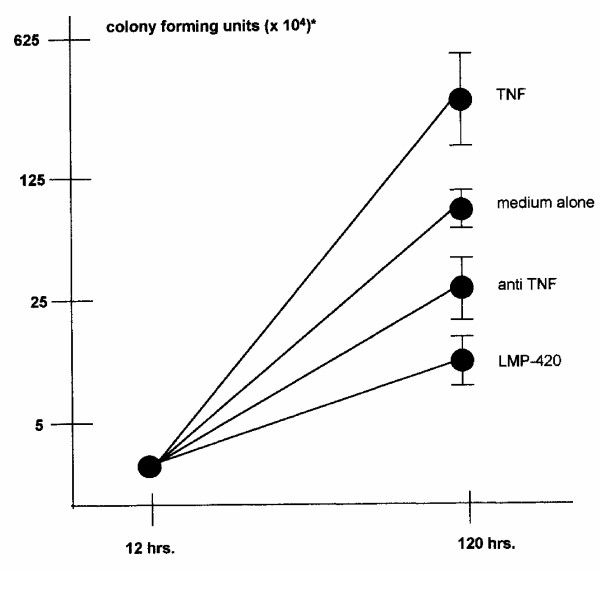
LMP-420 inhibits replication of *M. Tb *in human alveolar macrophages (AM). AM were collected by bronchial-alveolar lavage from healthy volunteers and infected overnight in culture with *M. Tb *(H37Rv; MOI = 1; efficiency of infection = 36 ± 9%). Cells (5 × 10^5^) were plated in 500 μl of medium in a 24-well plate supplemented with nothing, rhuTNF (10 ng/ml), anti-TNF (10 μg/ml) or LMP-420 (10 μM). The first time point for plating to determine CFU was taken immediately after the overnight infection. The second time point was taken after 120 h (108 h after treatment was initiated). Data represent the average ± SEM of thee experiments using 3 different donors.

Since a compound which could inhibit the release of TNF from both HIV and *M. Tb *would have added potential in areas where both pathogens are endemic, we first examined the ability of LMP-420 to inhibit TNF release from *M. Tb*-infected AM. AM derived from BAL were exposed to varying concentrations of LMP-420 for 2 h prior to being infected (MOI = 5) with *M. Tb*, the cells were cultured an additional 24 h, and TNF levels in the supernatants determined. LMP-420 inhibited TNF release from *M. Tb*-infected AM by 55, 68, and 90% at concentrations of 0.35, 3.5, and 35 μM respectively (data not shown). Since one of us (S. Stenger) had previously shown that anti-TNF antibody could block replication of virulent *M. Tb *[8], we now sought to determine if LMP-420 could likewise inhibit the replication of *M. Tb *in human AM. As shown in Figure 4, addition of 10 μM LMP-420 to cultures of human AM infected with a virulent strain of *M. Tb *(H37Rv; MOI = 1; efficiency of infection = 36 ± 9%) inhibits the replication of *M. Tb *by >80% over the subsequent 108 h of culture. The concentration of LMP-420 used (10 μM) would be expected to inhibit at least 70-80% of the *M. Tb*-stimulated TNF release under the conditions of these assays. Interestingly, the inhibition of replication by LMP-420 was even greater than that observed using 10 μg/ml of an anti-TNF polyclonal antibody which might suggest that LMP-420 inhibitory effects involve more than just TNF inhibition.

### LMP-420 is non-toxic to PBMC at the concentrations tested

Inhibition of the replication of either HIV or *M. Tb *by LMP-420 could arguably be the result of depletion of host cells by LMP-420. We first tested LMP-420 for toxicity in a MitoScan™ SMP assay looking at electron transfer with NADH [12]. No toxicity was observed with LMP-420 at concentrations up to 20 μM and the EC_50 _for LMP-420-mediated toxicity was 300 μM, a concentration that is 6000-fold greater than the EC_50 _for inhibition of TNF (Figure 1A). In addition to examining LMP-420's effects on mitochondrial function, three human lymphoid cell lines (CEM, lymphoblastoid; THP-1, monocytic; K562, erythroleukemic) were grown for 72 h in 50 μM LMP-420 with no observed effects on cell proliferation, as measured by [^3^H]-thymidine incorporation (data not shown). Further confirmation of the fact that LMP-420 is non-toxic are studies in which PBMC were stimulated for 72 h with 80 ng/ml of SEB in the presence or absence of either 2 μM or 0.4 μM LMP-420. Under these conditions LMP-420 inhibited TNF release by ~80% and ~60% respectively but had negligible effects on SEB-stimulated lymphocyte proliferation (~12% inhibition at 2 μM; 0% inhibition at 0.4 μM) (data not shown). Cumulatively, these results suggest that LMP-420's effects on either TNF release or the replication of HIV-1 or *M. Tb *are not due to cellular toxicity.

## Discussion

Co-infection with HIV and *M. Tb *is a major problem in developing countries. In addition to difficulties associated with simultaneous treatment of infections with two major pathogens, data suggests that each of these pathogens can enhance infection with the other. Numerous investigations have demonstrated that both HIV- and *M. Tb*-infected cells produce TNF and that either autocrine or paracrine produced TNF can enhance the replication of both pathogens.

Although *in vitro *studies such as those presented here suggest that targeting TNF to suppress replication of HIV and/or *M. Tb *might be a logical approach to enhance existing therapies, there are several reports of reactivation of latent *M. Tb *in patients being treated with TNF antagonist therapies [13,14]. The large majority of cases involving reactivation of latent *M. Tb *in patients treated with TNF antagonists appear to have occurred with infliximab (Remicade^™^; humanized monoclonal antibody) with ~144 and ~35 cases of tuberculosis per 100,000 patients occurring with infliximab and etanercept respectively from January 1998- September 2002 [15]. In a recent report of 12 cases of reactivation of latent *M. Tb *in patients in California being treated with TNF antagonists, 11 of the patients were being treated with infliximab [13]. Since infliximab is capable of binding to the transmembrane form of TNF and can subsequently activate complement, it is possible that infliximab might injure or kill TNF-expressing cells and thus function as a generalized immunosuppressive. Indeed, infliximab treatment in patients with Crohn's disease has been reported to induce apoptosis of both monocytes and T lymphocytes [16,17]. Although reactivation of latent *M. Tb *has also occurred with etanercept, it should be noted that a large proportion of patients being treated with these TNF antagonist agents are also being treated with other suppressive drugs such as methotrexate or steroids. In the study noted above [13], 8 of the 12 patients experiencing reactivation of latent *M. Tb *were being treated with prednisone, methotrexate or azathioprine.

An advantage of a small-molecule inhibitor of TNF transcription and release (such as LMP-420) over biologicals such as infliximab or etanercept is that such a molecule offers greater pharmacological control. The current TNF antagonists are designed to neutralize circulating TNF and are thus given at sufficiently high doses to maximize the time between injections which is dictated by the pharmacological half-lives of the molecules. By their nature, these antagonists neutralize essentially all of the circulating TNF and are irreversible. Although a small molecule will likely have a shorter half-life, this allows the intervention to be stopped should the clinical situation warrant it. Furthermore, LMP-420, while a very potent inhibitor of TNF release, inhibited only ~93% and 98% of TNF release from LPS-stimulated PBMC at 0.5 and 5.0 μM respectively (Figure 1A). The low level of TNF release which "escapes" inhibition by LMP-420 may be sufficient to maintain immune surveillance while LMP-420's inhibition of the majority of released TNF may protect against TNF-related pathogenesis. Nonetheless, since HIV+ patients are already immunosuppressed by their disease, more extensive testing, including clinical studies, will be necessary to determine whether treatment with a TNF biosynthesis inhibitor which is non-toxic and doesn't affect general cell function, such as LMP-420, might also reactivate latent *M. Tb*.

Our data confirms that LMP-420, an inhibitor of TNF transcription and subsequent biosynthesis, is able to inhibit the replication of both HIV-1 (Figure 2) and virulent *M. Tb *(Figure 4) in primary cultures of human cells. The doses at which LMP-420 inhibits replication of these pathogens are doses which have not been found to be toxic to either primary human leukocytes or human cell lines. Furthermore, LMP-420 has also recently been demonstrated to inhibit replication of *M. Tb *in human blood-derived dendritic cells [18]. Evidence doesn't suggest that LMP-420 has any direct anti-viral (G. Cianciolo, unpublished data) or anti-bacterial activity but rather, that it inhibits replication of these two pathogens by inhibiting the host cells' ability to support the pathogens' replication. In our studies we used an HIV strain (Ba-L) that is known to be monocyte/macrophage tropic. Whether LMP-420 would also affect T cell tropic strains of HIV remains to be determined although HIV-infected T cells also produce TNF [[19][20][21][22]] and LMP-420 inhibits TNF synthesis in T cells **(**Figure 1B). The dose at which LMP-420 inhibits 50% of HIV replication in PBMC is at least several-fold greater than the dose required to inhibit 50% of LPS-stimulated TNF release from PBMC. However, HIV replication is measured after 7 days of culture and LMP-420 was added only once, at the time of virus addition. Whether replenishing the LMP-420 during the 7 days of culture would lower the effective concentration of LMP-420 required for viral inhibition remains to be determined.

In 24-h cultures of LPS-stimulated PBMC (Table 1), 1.0 μM LMP-420 significantly inhibits the release of MCP-1 (95%) as well as TNF (91%). We have recently demonstrated by RT-PCR (G. Cianciolo, data not shown) that upregulation of mRNA for MCP-1 is completely blocked in 1.0 μM LMP-420-treated PBMC after 3 h of LPS stimulation. Nonetheless, we still cannot rule out the possibility that the inhibition of MCP-1 is secondary to the inhibition of TNF since studies in human airway epithelial cells have demonstrated strong TNF induction of MCP-1 mRNA within 1 h, the shortest time-period examined [23]. However, LMP-420 has recently been shown to also inhibit the upregulation of adhesion molecules (ICAM-1, VCAM-1, CD40) on human brain-derived endothelial cells activated by either TNF or lymphotoxin (LT; TNF-beta) and to prevent the release of microparticles (a sign of inflammation) from such activated cells [24]. Regardless of the mechanism of MCP-1 inhibition by LMP-420, blockade of this chemokine is potentially advantageous since MCP-1 has been demonstrated to enhance HIV replication [25]. Although the role of MCP-1 in the replication of *M. Tb *is less clear, a recent study demonstrated that co-infection of macaques with simian immunodeficiency virus (SIV) and *Mycobacterium avium *complex (MAC) significantly increased levels of MCP-1 in both serum and tissue samples [26].

## Conclusion

The cell culture studies presented here confirm that strategies designed to inhibit the release of pro-inflammatory cytokines/chemokines, such as TNF and MCP-1, may be beneficial in inhibiting the replication of HIV-1, *M. Tb*, or both. The potential advantages of small-molecule, inexpensive, stable compounds over existing biologicals in areas where both pathogens are endemic suggest that this approach is deserving of further investigation.

## Methods

### Reagents

LMP-420, 2-NH_2_-6-Cl-9- [(5-dihydroxyboryl)-pentyl] purine, a gift from LeukoMed, Inc. (Raleigh, NC), was stored as a 10 mM stock solution in dimethylsulfoxide (DMSO; Sigma-Aldrich, St. Louis, MO). Anti-CD3 (OKT3) antibody, staphylococcus enterotoxin B (SEB), and lipopolysaccharide (LPS) were obtained from Ortho Biotech (Bridgewater, NJ), Calbiochem (San Diego, CA), and Sigma-Aldrich, respectively.

### Separation and stimulation of PBMC

PBMC were separated from buffy-coated blood of healthy donors by standard Ficoll-Hypaque gradient centrifugation procedures. PBMC were activated with 5 μg/ml of phytohemagglutinin (PHA) plus 10 ng/ml of IL-2 in RPMI 1640 culture medium with L-glutamine (2 mM), supplemented with 20% fetal bovine serum and 100 U/ml penicillin and 100 μg/ml streptomycin (growth medium) for 2- 3 days at 37°C in 5% CO_2 _and 95% humidified air.

### Infection of stimulated PBMC with HIV-1Ba-L

Activated PBMC were washed and infected with HIV-1_Ba-L _(NIH AIDS Research & Reference Reagent Program, Germantown, MD) at 500 × TCID_50 _determined by previous propagation in normal PBMC for 5- 7 days. HIV-1_Ba-L_-infected PBMC were incubated at 37°C in 5% CO_2 _and 95% humidified air for 2 h, mixed by gentle swirling every 20- 30 min, and centrifuged for 10 min at 200 g at room temperature. Cell pellets were gently resuspended in growth medium.

### Treatment of infected cells with LMP-420 and/or AZT

HIV-1_Ba-L _infected PBMC (1 × 10^6^/ml/well) were treated with or without drug (LMP-420 and/or AZT) with equivalent amounts of DMSO in a 48-well plate at 37°C in 5% CO_2 _and 95% humidified air for 7 days. Compounds were not cytotoxic at the concentrations used during the assays and viability of infected and treated cells, as measured by trypan blue, was >85%.

### Titration of HIV-1 p24 antigen

Supernatants were collected at 7 days post-infection and viral replication quantitated by measurement of the HIV-1 specific core antigen, p24, by radioimmunoassay, using the protocol provided by the supplier (Beckman Coulter, Hialeah, FL).

### Infection of AM with M. Tb

Human AM were obtained and infected with *M. Tb *as previously described [8]. Briefly, AM were obtained from the discarded bronchoalveolar lavage (BAL) fluid of patients who had undergone bronchoscopy for diagnostic purposes after all identifiers had been removed. Purity (>95%) of the cells was confirmed by α-naphthyl-acetate esterase staining (Sigma-Aldrich) and flow cytometric analysis (CD3 <1%, CD19, CD56, CD66, CD1 negative). Viability was >96% as determined by trypan blue dye exclusion.

AM were infected with single cell suspensions of *M. Tb *in six-well culture plates at 1 × 10^6 ^cells/ml in a final volume of 3 ml. After 4-h incubation at 37°C, extracellular bacteria were removed by intensive rinsing with PBS. To quantitate mycobacterial growth, the adherent cells were harvested by gentle scraping with a cell scraper and re-plated at a concentration of 1 × 10^6 ^cells/ml in a 24-well plate (final volume 500 μl) in complete medium without antibiotics plus 10% human serum. Cell viability of infected AM was determined by trypan blue exclusion and was >99%.

For determination of CFU, infected cells were lysed with 0.3% saponin (Sigma-Aldrich) to release intracellular bacteria. At all time points an aliquot of un-lysed, infected cells was harvested and counted, allowing an exact quantification of cells as well as determination of cell viability. Recovery of cells was >80% in all experiments, with cell viability regularly >90%. CFUs of lysates were determined as previously described [8].

### Toxicity assays for LMP-420

General toxicity of LMP-420 was evaluated using the MitoScan™ SMP assay (submitochondrial particle; Harvard Biosciences, Holliston, MA) per the manufacturer's instructions. Verapamil-HCl (Sigma-Aldrich) was used as an internal positive control (EC_50_~150 μM). Compounds were prepared as a 338 mM stock (LMP-420) or a 285 mM stock (verapamil-HCl) in DMSO and the highest concentration of DMSO in the assays was 0.7%. MitoScan™ provides a rapid, homogeneous assay that correlates highly with cell proliferation assays, cell viability assays and other cytotoxicity or apoptosis endpoints such as MTT, LDH and Alamar Blue assays. In addition to its effects on mitochondrial activity, LMP-420 was tested for growth-inhibitory effects on three human lymphoid cell lines (CEM, THP-1, K-562; ATCC, Manassas, VA). To each of duplicate sets of 6 wells of a 96-well cell culture plate was added 5 × 10^3 ^of each cell type. One set was cultured for 72 h in media alone and the other was cultured in 50 μM LMP-420. For the last 4 h of culture, 0.5 μCi of [^3^H]-thymidine (6.7 Ci/mmole; New England Nuclear-PerkinElmer Life and Analytical Sciences, Boston, MA) was added to each well and the contents of each well harvested onto glass fiber filters and incorporated radioactivity determined by liquid scintillation spectrophotometry.

## Abbreviations

AM, alveolar macrophage; BAL, bronchoalveolar lavage; CFU, colony forming units; HIV, human immunodeficiency virus type 1; LPS, lipopolysaccharides; LTR, long terminal repeat; NO, nitric oxide; PBMC, peripheral blood mononuclear cells; PHA, phytohemagglutinin; rhIL-2, recombinant human interleukin-2; SEB, staphylococcus enterotoxin B; TNF, tumor necrosis factor-alpha;

## Competing interests

The author(s) declare that they have no competing interests.

## Authors' contributions

SH, GJC, NKD and SS designed the experiments. SH, GJC, WK and MBP performed the experiments. SH, GJC, WK, NKD, NT, MBP and SS analyzed the data. SH, GJC, NKD, WK, MBP, SS, NT, JWS and SP wrote, edited, and reviewed the manuscript.
